# Why are some racial disparities harder to interpret than others?

**DOI:** 10.3389/fsoc.2026.1798604

**Published:** 2026-07-09

**Authors:** Rima Wilkes, Aryan Karimi

**Affiliations:** Department of Sociology, University of British Columbia, Vancouver, BC, Canada

**Keywords:** Black–Asian, Black–White, cultural differences, dyadic comparison, normative interpretive strain, racial disparities, racial inequality, structural racism

## Abstract

By definition, every racial disparity entails an ordering of racial groups, with one positioned higher and another lower. Cultural frameworks typically interpret disparities by emphasizing higher outcome positions as desirable, while implicitly casting lower positions as requiring improvement. Structural frameworks, by contrast, interpret disparities by treating higher outcome positions as structurally advantaged and therefore as the locus of critique. This article proposes that each framework contains an implicit evaluative contrast that problematizes one side of the disparity either as “deficient” or as “advantaged.” We introduce the concept of “normative interpretive strain” to describe the tension that emerges when empirical orderings place particular racial groups into positions carrying difficult or negative evaluative meanings. Using Asian, Black, and White comparisons in the U.S. context, we argue that, within cultural frameworks, normative interpretive strain emerges primarily when Black Americans occupy lower outcome positions associated with deficiency-oriented interpretations. Within structural frameworks, normative interpretive strain emerges when minority groups occupy positions associated with structural advantage and when White Americans no longer consistently occupy advantaged positions. The analysis proceeds in two stages. Part 1 examines dyadic comparisons, showing that Black-White disparities generate normative interpretive strain for cultural explanations while White-Asian disparities generate normative interpretive strain for structural explanations. Both frameworks encounter normative interpretive strain in Black-Asian comparisons. Part 2 extends the analysis to triadic comparison by examining outcome rankings among Asian, Black, and White populations across socioeconomic and health domains. Normative interpretive strain becomes more visibly apparent once multiple contrasts involving the same groups must be accommodated simultaneously. The analysis further shows how heterogeneity, selectivity, and multiplicity arguments operate as partial “repair” strategies for managing normative interpretive strain. By shifting attention from causal explanation to *a priori* meaning assignment to group rankings, the article maintains that normative interpretive strain is not an artifact of anomalous data or selective outcomes. Rather, it is an inherent feature of how racial disparities are rendered sociologically intelligible.

## 1 Introduction

How is the term “racial disparity” generally understood? At a first pass, a racial disparity is a numerical gap between how two racial groups experience life in terms of poverty, health status, and education, among other aspects. However, not all disparity outcomes are equally interpreted or researched as racial disparities. Some disparities, such as those between Black and White populations are routinely treated as symptoms of inequality (Choper, 2025; Boen, 2016; Williams and Sternthal, 2010). Other disparities, such as those between White and Asian populations, are sometimes treated as ambiguous findings thereby requiring further interpretive explanations. This further interpretation might entail qualification of the disparity, contextualization, or a shift in analytic emphasis (cf. Sakamoto, 2017, 2025 on this point). This pattern is noteworthy. Although racial disparities are descriptive numerical differences, they can carry implicit evaluative meanings about both the outcome and the racial groups to whom they apply (Jung, 2009; Bonilla-Silva, 2011; Iceland et al., 2025). Such differences in interpretive treatment are well recognized. Still, the bulk of research on racial disparities accounts for the causes of disparities. The meanings attributed to disparity outcomes themselves are rarely analyzed as a phenomenon in their own right.

One set of causal accounts posits that disparities emerge from shared or divergent cultural values, norms, and practices (Murray, 1984; Beugelsdijk and Klasing, 2016). Early formulations emphasized values and behavior transmission across generations (Auletta, 1982; Lewis, 1966). Later accounts framed culture as a set of adaptive responses to social and economic conditions rather than as fixed traits (Small et al., 2010). As an analytic orientation, cultural frameworks render disparities legible by comparing groups along dimensions of relative performance, including variation in work ethic, educational investment, parental expectations, and family orientation (Farkas et al., 1990; Hsin and Xie, 2014; Sakamoto et al., 2021). The normative evaluative meaning of disparity outcomes, under cultural explanations, thus tends to take the form of a deficient-meritorious contrast, or some variant thereof. Under this contrast groups occupying the top outcome position are relatively valorized while those positioned at the bottom are implied to be deficient or in need of improvement (cf. Russell et al., 2022 on this point).

A second set of causal accounts examine racial disparities as outcomes of structural group-based power relations that shape life chances independently of individual behavior (Tilly, 1998; Weeden and Grusky, 2012; Wright, 1997). Within this framework, unequal outcomes are attributed to macro-level processes such as residential segregation, labor market discrimination, and differential access to resources (Bonilla-Silva, 1997; Christian, 2019; Rugh and Massey, 2010). As an analytic orientation, structural frameworks render disparities legible by situating group outcomes within hierarchical systems of constraint and advantage. The normative evaluative logic of structural explanations is organized around a disadvantaged-advantaged contrast. By treating advantage as structurally produced, while interpreting lower outcome positions as reflecting systemic constraint the disadvantaged-advantaged contrast problematizes groups occupying the top outcome position (Feagin and Elias, 2013; Bonilla-Silva, 1997).

In both examples, the evaluative meanings affixed to the sides of disparity gaps are analytically distinct from the causal processes used to explain them. The specific meanings attached to higher and lower group positions are themselves historically, politically, and institutionally shaped rather than analytically neutral (Bonilla-Silva, 2011; Iceland et al., 2025). This paper examines how these meanings structure the interpretation of racial disparities once particular empirical group orderings are observed.

To make the case, the paper proceeds in two stages. Part 1, based on 2023–2024 U.S. Census data (Koller and Scherer, 2025; Shrider, 2024; U.S. Census Bureau, US Department or Commerce 202 AD, 2022) examines racial disparities dyadically. First we focus on the Black-White poverty gap as a canonical case in U.S. racial inequality research (cf. Brady, 2023; Baker et al., 2022). Next, to provide further examples for the frameworks’ interpretive work, we add the Black-White education and income gaps (Daniels et al., 2024; Hardy et al., 2025), and then Asian-White, White-Asian and Black-Asian disparity gaps (Dunatchik and Park, 2022; Hsin and Xie, 2014).^1^ Part 2 uses the Census data to extend the analysis beyond dyads to triadic comparisons. Here we consider triadic outcome rankings among Black, White, and Asian populations on poverty, education and income. We also consider a wide range of other socioeconomic, health and political domains. Rather than arranging the comparisons according to the measures or group names, we follow the empirical data to comparatively rank order the disparities along a low-high numerical continuum.

The analysis overall shows that certain disparities, such as White-Asian disparities within cultural frameworks and Black-White disparities within structural frameworks, align not simply because they are causally compatible with those frameworks. Rather, the empirical ordering places racial groups into evaluative positions that are normatively admissible within them. Other disparities, however, require additional interpretive work to be accommodated^2^. When Black-White disparities repeatedly place Black Americans at the lower end of outcome distributions, cultural explanations encounter interpretive strain. This lower end position is linked to a “deficit” position, which is normatively inadmissible, while structural explanations remain relatively compatible. In contrast, when White-Asian disparities place Asian Americans at higher outcome positions, cultural explanations stabilize. Asian at the higher position can be described as meritorious, while structural explanations encounter interpretive strain since it is unprecedented for these frameworks to assign advantage to a racial minority group or disadvantage to the racial majority group (cf Wu et al., 2022).

We propose the concept of “normative interpretive strain” to describe the strain that emerges when empirical outcomes place particular racial groups into the negatively or positively valued positions embedded within explanatory frameworks. Both frameworks encounter normative interpretive strain in Black-Asian comparisons, where, as the data show, Black Americans are positioned as “deficient” under cultural explanations and Asian Americans are positioned as “advantaged” under structural ones. In both cases, normative interpretive strain emerges out of concern for “the welfare of protected groups” (cf Martin, 2016: 118). Attending to how cultural and structural frameworks differently evaluate the lower and higher positions within a disparity therefore helps explain why some racial disparities fit more smoothly within particular frameworks than others.

In this sense, every disparity comparison implicitly positions at least one racial group as “the problem,” either as deficient within cultural explanations or as advantaged within structural explanations. Du Bois (1903) observed this dynamic in relation to Black Americans through the question, “How does it feel to be a problem?”. We underline this insight by arguing that such problematization is a structural feature of comparative racial analysis itself. That is, to study racial disparity depends on an evaluative logic (e.g., in cultural and structural theories) and the empirical rank-ordering of the groups according to the normative evaluation. We then engage with recent debates that address this group-problematization issue: heterogeneity (subdivides the groups), compositional selectivity (adjusts the comparison), and multiplicity (allows group to occupy multiple positions). These strategies temporarily stabilize interpretation, but they cannot fully resolve the underlying tension. Comparative analysis itself requires that at least one racial group occupy a side of a disparity that already carries negative connotations.

### 1.1 Part 1. Dyadic racial comparisons: evaluative meanings and normative interpretive strains

This section outlines how cultural and structural frameworks assign evaluative meaning in dyadic racial comparisons. While the aim of both frameworks is theoretical description it is also the case that “they often carry normative assumptions” (Iceland, 2025: 922). We begin with the Black-White poverty disparity, a canonical case in U. S. racial inequality research (Iceland, 2019; Williams and Baker, 2021). Our aim is not to adjudicate the causes of this poverty disparity in this paper, but to make explicit the frameworks’ interpretive meanings through which a widely accepted racial disparity is often rendered intelligible. Using this case, we formalize the distinct evaluative grammars embedded in cultural and structural explanations (Herring and Henderson, 2016). We show how the same empirical ordering acquires different positional meanings, in cultural and structural frameworks, *prior* to causal attribution. We then extend the analysis to Black-White educational and income outcomes. The repeated placement of the same groups on higher and lower outcome positions generates cumulative interpretive consequences within each framework. Finally, we apply these same evaluative grammars to disparities entailing Asian Americans. The resulting change in group ordering when Asian Americans are added produces interpretive stability for cultural explanations but normative interpretive strain for structural ones. Taken together, these dyadic analyses establish that interpretive coherence is not an inherent property of particular frameworks or outcomes. Rather it depends on how empirical orderings align with framework-specific meaning structures.

Cultural explanations emphasize individual-level values and behaviors as sources of disparate outcomes. They rest on the assumption that those at the bottom of the social and economic hierarchy can catch up to those with better outcomes by adopting a “minority culture of mobility” centered on hard work, thrift, education, and the accumulation of social and cultural capital (Neckerman et al., 1999 p. 946; see also Banfield, 1970).^3^ Within this framework, poverty disparities are generally interpreted through an implicit deficient-meritorious contrast, with Black and White Americans occupying opposing evaluative positions.

Over time, cultural approaches to the racial poverty gap had to contend with two recurring problems. First, the framework had to explicitly explain the role of racism and discrimination in disparities. Second, the focus on deficits in schooling, family and spending, positions the lower pole of the disparity as the location requiring amelioration. This implies that the Black group is “the problem needing to ‘catch up’ to whites” (Seamster and Ray, 2018, p. 325), mirroring Du Bois’ (1903) observation that Black people were not seen as individuals but rather as a burden or problem to be “ignored, pitied, or stigmatized” (see Barnes, 2003, p. 1).

Structural accounts of the Black-White poverty disparity address this limitation of cultural frameworks through an alternative evaluative approach. Within this framework, occupying a disadvantaged social location is understood as carrying significant social, political, and material constraints. Structural accounts direct attention to the macro and structural level. Individuals at the bottom of the social hierarchy are understood as systematically disadvantaged by a system that favors the members of some social groups over others. Racism, conceptualized as a system of group domination is “embedded and operating in corporations, universities, legal systems, political bodies, cultural life, and other social collectives” (Desmond and Emirbayer, 2009, p. 345).

Theories derived from structural logics attribute racial disparities in poverty to institutional barriers such as residential segregation (Massey and Denton, 1993), labor market discrimination (Pager and Shepherd, 2008), unequal schooling (Pattillo, 2005), and mass incarceration (Alexander, 2010). These barriers systematically constrain opportunities for marginalized racial groups. Rather than behavioral variables, factors such as “access to credit”, “retail and service desertification” and “consumer discrimination” explain the socioeconomic differences between Black and White groups (Charron-Chénier et al., 2017, p. 2). These structural constraints are maintained through policies and practices that reproduce economic disadvantage across generations, independent of individual effort or cultural values (Oliver and Shapiro, 1995). In effect racialized policies “concentrated poverty, undermined mobility out of poverty, and exacerbated the interaction of race and poverty” (Brady, 2023, p. 13).

The focus on the macro-level systems and institutions might create the impression that structural theories do not have a problematization-valorization system of meanings assigned to groups. However, the evaluative interpretations are not removed so much as reconfigured through a different contrast. Whereas cultural theories frame the evaluative distinction in terms of deficiency and merit, structural theories organize interpretation around a disadvantaged versus advantaged contrast. Within this framework, analytic attention is directed toward how advantage is produced and reproduced (Jardina and Piston, 2021; Leonardo, 2004; Weisenburger, 2002).^4^ In this formulation, disadvantage functions as the normatively focal position and advantage as the primary site of analytic critique (cf. Lamont, 2002). The empirical pattern that cultural accounts interpreted through the deficient and meritorious contrast is reinterpreted through the disadvantaged and advantaged contrast. The empirical ordering remains the same, while the evaluative meaning attached to each side is inverted. It is a not a moral positive to be “advantaged” under the structural framework (Silver et al., 2026).

The analysis of the Black-White poverty disparity shows that racial disparities are not neutral outcomes awaiting explanation. Empirical disparities position groups along axes that carry evaluative meanings prior to causal attribution. Cultural and structural explanations organize these evaluative contrasts differently. Table 1, based on the Black-White poverty gap, formalizes how cultural and structural explanations assign evaluative meaning to higher and lower outcome positions and how these assignments condition interpretive fit.

**Table 1:** Cultural and structural interpetive meaning systems assigned to groups on either side of racial disparity outcomes.

Observation of a disparity	(Earlier) cultural paradigm’s implicit relational evaluative implication		(Current) structural paradigm’s implicit relational evaluative implication)	
Poverty: Black–White	**Deficit:** high levels of poverty (among Black) reflect a lack in relative performance, orientation, or practices	**Meritorious:** low levels of poverty (among white) reflect appropriate performance, orientation, or practices	**Disadvantaged:** high levels of poverty (among Black) reflect structural constraint or unequal position	**Advantaged:** low levels of poverty (among white) reflect structural position or accumulated benefit

This table formalizes the interpretive grammar through which explanatory frameworks assign meaning to observed outcome positions. The specific labels are illustrative; alternative terms would serve the same analytic purpose. Table 2 shows that recent cultural frameworks have explicitly rejected this labelling of Black–White disparities.

Table 1 shows that within cultural approaches, outcome positions tend to be read through a deficient-meritorious contrast that foregrounds effort, behavior, and performance. Relatively better outcomes are more readily taken as evidence of merit, while relatively poorer outcomes come to appear problematic and in need of explanation. Structural approaches orient interpretation in the opposite direction: advantaged positions as reflect structural benefit and inferior outcomes as reflect systemic constraint. Table 2 extends this insight by showing the link between these basic evaluative meanings and further empirical evidence for Black-White racial disparities.

**Table 2:** Cultural and structural paradigms’ meaning-system assigned to groups on either side of racial disparity outcomes—further examples.

Observation of a disparity:	(Earlier) cultural paradigm’s implicit meanings:		(Current) structural paradigm’s implicit meanings:	
	**Deficit:** high levels of poverty reflect a lack in relative performance, orientation, or practices	**Meritorious:** better outcomes reflect appropriate performance, orientation, or practices	**Disadvantaged:** poor outcomes reflect structural constraint or unequal position	**Advantaged:** better outcomes reflect structural position or accumulated benefit
Poverty %, 2023: Black (17.9) - White (7.7)	Normative interpretive strain (position implies Black “deficit”)		Compatible	
Education % (college grad), 2023: Black (27.0) - White (40.0)				
Income, median 2024: Black ($56,020) - White ($88,010)				

Earlier cultural frameworks deficit-meritorious contrast led to strain as a result of the implication that, based on the empirical ordering, Black is deficient. Structural theories do not encouter this strain in relation to this contrast.

Table 2 links observed empirical outcome patterns to the evaluative contrasts outlined in Table 1. Whereas Table 1 specifies how cultural and structural frameworks assign evaluative meaning to higher and lower outcome positions, Table 2 demonstrates how these evaluative grammars are activated when applied to empirical data on racial disparities. Using poverty, educational attainment, and income as illustrative domains, Table 2 shows how identical empirical orderings are organized and interpreted differently depending on the explanatory framework employed. Empirically, Black Americans exhibit higher poverty rates than White Americans, with approximately 17.9 percent of Black individuals living in poverty compared to 7.7 percent of White individuals. In educational attainment, 17.9 percent of Black adults hold a college degree, compared to 40.0 percent of White adults. Income disparities follow the same ordering, with median household income for Black Americans at approximately $56,020, compared to $88,010 for White Americans.^5^ Across these domains, Black Americans consistently occupy the lower outcome position, while White Americans occupy the higher outcome position.

Within (earlier) cultural frameworks, Black Americans’ higher poverty rates and lower levels of educational attainment and income are more readily interpreted as outcomes requiring explanation, often framed in terms of individual behavior. White Americans’ comparatively advantaged outcomes, by contrast, are treated as unremarkable or implicitly attributed to individual merit.^6^ Subsequently, because it assigns negative evaluative meaning to lower outcome positions and positive evaluative meaning to higher ones the framework faces normative interpretive strain since it risks reinforcing negative interpretations of Black Americans while valorizing White Americans.

In comparison, structural frameworks organize the same empirical evidence through a disadvantaged–advantaged contrast. Black Americans’ higher poverty rates and lower levels of education and income are interpreted as the product of systemic constraint. White Americans’ higher outcome positions are understood as reflecting structural benefit rather than individual merit. This evaluative configuration is admissible because it aligns with historical and institutional analyses and avoids attributing group differences to intrinsic or behavioral deficiencies. The relative interpretive appeal of structural explanations in Black-White comparisons derives not solely from their causal claims, but from the evaluative meanings they attach to groups on either side of the empirical disparity gaps.

The evaluative strain and admissibility of the cultural and structural frameworks, however, are contingent on specific empirical configuration examined. As the following sections show, extending the analysis beyond Black-White dyads and beyond outcomes that conform to familiar hierarchies, introduces new forms of framework-level interpretive strain. Table 3 holds the same three outcomes —poverty, education and income—constant and shows what happens when Asian Americans are included.

**Table 3:** Empirical evidence generates normative interpretive strain where implicit meanings do not correspond to (anticipated) groups.

Observation of a disparity	Earlier/current cultural explanations		Current structural explanations	
	**Deficit:** high levels of poverty reflect a lack in relative performance, orientation, or practices	**Meritorious:** better outcomes reflect appropriate performance, orientation, or practices	**Disadvantaged:** poor outcomes reflect structural constraint or unequal position	**Advantaged:** better outcomes reflect structural position or accumulated benefit
Poverty %, 2023: Black (17.9) - White (7.7)	Strained (position implies Black “deficit”)		Compatible	
Education % (college grad), 2023: Black (27.0) - White (40.0)				
Income median, 2024: Black ($56,020) - White ($88,010)				
Poverty %, 2023: Asian (9.1) - White (7.7)	Compatible		Compatible	
Education % (college grad), 2023: White (40) - Asian (57.8)			Strained (position implies White “disadvantage”)	
Income median, 2024: White ($88,010) - Asian ($121,700)				
Poverty %, 2023: Black (17.9) - Asian (9.1)	Strained (position implies Black “deficit”)		Strained (position implies Asian “advantage”)	
Education % (college grad), 2023: Black (27.0) - Asian (57.8)				
Income median, 2024: Black ($56,020) - Asian ($121,000)				

Normative interpretive strain is engendered within each paradigm for different reasons. Earlier cultural explanations applied to Black-White, while current cultural explanations are typically applied to Asian-White. Empirical contrasts that place Black on the poorer outcome side imply a relative deficit meaning thereby invoking normative strain for cultural explanations. Empirical contrasts that place POC as advantaged and White as relatively disadvantaged will engender strain for structural explanations.

Table 3, based on the same three outcomes of poverty, educational attainment, and income, examines how interpretive dynamics change when Asian Americans are incorporated alongside Black and White Americans. Empirically, Asian Americans outperform White Americans in education and income, while exhibiting a higher poverty rate. Black Americans, by contrast, occupy the lowest outcome position across all three domains. This tri-racial configuration allows for a direct comparison of how evaluative assignments shift when the group occupying the lowest position is Black versus when relative ordering varies between Asian and White Americans.

Within cultural frameworks, explanations that link poor outcomes to individual behavior or cultural traits are widely regarded as normatively problematic when applied to Black Americans. Table 3 shows that this constraint does not extend symmetrically to other racial comparisons. When Asian and White Americans are compared, cultural frameworks display considerable evaluative flexibility. Asian Americans’ higher poverty rate relative to White Americans does not generate the same normative interpretive strain that would arise if similar reasoning were applied to Black Americans. Instead, cultural explanations accommodate this pattern by treating Asian poverty as contingent or transitional, as in assimilation frameworks (Takei and Sakamoto, 2011). Asian Americans’ superior outcomes in education and income are also readily interpreted in cultural accounts that emphasize exemplary traits such as work ethic, educational investment, and family orientation (Dunatchik and Park, 2022; Farkas et al., 1990; Hsin and Xie, 2014). Within these accounts White Americans are implied or described “just alright” (Jiménez and Horowitz, 2013). The key point is not whether Asian Americans are ultimately classified as meritorious or deficient in any single domain, but that cultural frameworks permit either classification without destabilizing the framework itself.

Structural frameworks exhibit a different pattern. Structural explanations remain normatively stable when Black Americans occupy the disadvantaged position and White the advantaged one. The placement of Asian in the advantaged category on education and income then creates normative interpretive strain (cf Martin and Nezlek, 2014) on Asian income under-estimation). Assigning advantage to a racial minority group and disadvantage to a racial majority group challenges the conventional linkage between advantage and historical dominance. Then, it appears there is a plausible division of analytic labor in which cultural explanations explain White-Asian or Asian-White disparities and structural explanations explain Black-White disparities. The final rows of Table 3 suggest that when Black and Asian Americans are compared directly, however, both frameworks encounter interpretive difficulty. Cultural accounts cannot readily attribute Black disadvantage to deficiency without violating normative constraints. Structural accounts struggle to explain Asian advantage without severing the link between advantage and historical dominance. Table 3 thus demonstrates that interpretive stability as well as interpretive strain in racial disparity analysis is not a property of particular frameworks alone, but of specific empirical configurations that allow certain contrasts to remain foregrounded while others are analytically qualified.

### 1.2 Part 2. Triadic racial comparisons: evaluative meanings and normative interpretive strains

While these theories typically also entail the combination of multiple axes, here, in Table 4, we consider three groups along single axes.

**Table 4:** In the triadic case normative interpretive strain becomes systematic- in addition to position the absolute rate on each outcome will locate the middle group closer to one or the other side.

Observation of a disparity				Earlier/current cultural explanations		Current structural explanations	
				**Deficit:** high levels of poverty reflect a lack in relative performance, orientation, or practices	**Meritorious:** better outcomes reflect appropriate performance, orientation, or practices	**Disadvantaged:** poor outcomes reflect structural constraint or unequal position	**Advantaged:** better outcomes reflect structural position or accumulated benefit
Poverty %, 2023	Black (17.9)	Asian (9.1)	White (7.7)	Strained (position implies Black “deficit”)		Strained (rate pushes Asian into “advantage”)	
Education % (college grad), 2023	Black (17.9)	White (40)	Asian (57.8)			Strained (position implies Asian “advantage”)	
Income median, 2024	Black ($56,020)	White ($88,010)	Asian ($121,700)			Strained (rate pushes White into “disadvantage”)	
Poverty children %, 2023	Black (25.0)	Asian (11.6)	White (8.7)	Strained (position implies Black "deficit")		Strained (rate pushes Asian into "advantage")	
Home ownership %, 2024	Black (45.8)	Asian (62.5)	White (74.2)				
Usual place of health care %, 2024	Black (85.5)	Asian (87.8)	White (89.8)				
Wealth median net worth, 2022	Black ($31,250)	White ($256,000)	Asian ($383,800)	Strained (position implies Black "deficit")		Strained (position implies Asian "advantage" & rate on some outcomes pushes White into "disadvantage")	
Manag/prof occ. %, 2024	Black (36.2)	White (44.1)	Asian (58.9)				
Unemployment rate %, 2023	Black (5.5)	White (3.3)	Asian (3.0)				
No health insurance, % 2024	Black (9.7)	White (6.5)	Asian (5.8)				
Life expect. at birth years, 2023	Black (74.0)	White (78.4)	Asian (85.2)				
All cause mortality, rate per 100 K, 2023/24	Black (924.4)	White (778.4)	Asian (387.9)				
Incarc. per 100 K, 2012–2022	Black (1,826)	White (337)	Asian (141)				
Imprisonment per 100 K, 2012–2022	Black (911)	White (188)	Asian (71)				
Serious psychological distress, % 2023	White (14.2)	Black (11.4)	Asian (10.2)	Compatible		Strained	
Smoking during pregnancy %, 2023	White (4.4)	Black (2.7)	Asian (0.2)	Strained (rate pushes Black into “deficit”)		(position implies White “disadvantage” and Asian “advantage”)	
Mortality from unintentional injuries %, 2019	White (23.7)	Black (19.9)	Asian (12)				
National belonging where are you from? %, 2021	Asian (64)	Black (27)	White (7)	Compatible		Strained (rate pushes Black into "advantage")	
Voter turnout %, 2020	Asian (60)	Black (63)	White (71)	Strained (rate pushes Black into "deficit")		Compatible	
Senior executive service rep/l.f. share %, 2018	Asian (3.8)	Black (10.6)	White (78.8)	Compatible		Compatible	
Self-esteem effect sizes, 2002	Asian (−0.30)	White (0)	Black (0.19)	Compatible		Strained (position implies Black "advantage")	
Union representation %, 2024	Asian (9.8)	White (10.8)	Black (13.2)				

The table summarizes triadic outcome rankings for Black, White, and Asian populations across multiple domains. Indicators differ in whether higher or lower values are positively valued. Triadic orderings generating normative interpretive strain recur across outcome domains even with different relative group ranking and rates, indicating that the analytic issues identified earlier are not artifacts of selective outcome choice.

Table 4 begins by holding constant the same three outcome domains examined previously, poverty, educational attainment, and income, and then applying the same cultural and structural evaluative grammars but at a triadic level. In the triadic poverty distribution, Black Americans exhibit the highest poverty rate at 17.9 percent, followed by Asian Americans at 9.1 percent and White Americans at 7.7 percent. This ordering immediately generates normative interpretive strain for cultural frameworks by implying deficit. Structural frameworks, by contrast, initially appear unstrained. The ordinal ordering preserves White advantage and Black disadvantage, aligning cleanly with expectations rooted in historical dominance and exclusion.

However, this compatibility dissolves once a more nuanced triadic comparison introduces rate-based evaluation. Asian Americans’ poverty rate of 9.1 percent is far closer to the White rate of 7.7 percent than to the Black rate of 17.9 percent. This proximity puts Asian Americans in the same grouping with White Americans on the advantaged side of the distribution rather than treated as an intermediate category. Structural strain emerges because the advantaged cluster now consists of a historically subordinate group paired with the historically dominant one. Thus, what appears structurally acceptable on an ordinal reading becomes strained once absolute rates are taken seriously, revealing that triadic comparison destabilizes structural interpretation even in domains where rank initially appears favorable.

Education and income produce an outcome configuration that immediately leads to normative interpretive strain for both cultural and structural frameworks, though for distinct reasons. In educational attainment, 17.9 percent of Black Americans hold a college degree, compared to 40.0 percent of White Americans and 57.8 percent of Asian Americans. In income, median earnings are $56,020 for Black Americans, $88,010 for White Americans, and $121,700 for Asian Americans. From a cultural perspective, the placement of Black Americans at the bottom of both distributions generates strain because it implicitly frames disadvantage in terms of deficit. Cultural explanations are pressured to account for why Black Americans consistently occupy the lowest position, thereby reintroducing the very interpretive logic they seek to avoid.

Structural frameworks encounter a different but equally immediate normative interpretive strain. In both domains, the group occupying the highest position is Asian Americans rather than White Americans, undermining the expectation that historical dominance anchors in advantage. Triadic comparison then intensifies this strain by introducing rate-based evaluation. The distance between Asian and White outcomes is substantial, particularly in income, where Asian Americans earn over $33,000 more than White Americans, while the White-Black income gap is approximately $32,000. This alignment places White Americans somewhat closer to Black Americans than to Asian Americans, pushing White Americans toward the disadvantaged side of the distribution rather than stabilizing them as an intermediate category. Structural frameworks must therefore either accept a decoupling of dominance from advantage or introduce additional qualifications to preserve their explanatory coherence. In this way, triadic comparison transforms positional inconsistency into amplified evaluative strain by forcing frameworks to reckon not only with rank but with the magnitude of group separation. Next, Table 4 adds other outcome measures.

The next two sets of outcomes reproduce the same interpretive dynamics observed in poverty, education, and income, while differing in ordinal configuration. The first set, which includes child poverty, unemployment, and homeownership, follows a Black-Asian-White ordering. Black Americans occupy the most disadvantaged position, Asian Americans are in the middle, and White Americans are the most advantaged. This configuration generates cultural strain through the repeated placement of Black Americans at the bottom of the distribution, implicitly invoking deficit-based interpretation across multiple domains. Structural strain emerges once triadic comparison introduces rate-based evaluation. For example, in child poverty, Black Americans experience a rate of approximately 27 percent, compared to about 11 percent for Asian Americans and 9 percent for White Americans. Although Asian Americans occupy the middle rank, their rate is far closer to White American advantage than to Black American disadvantage, encouraging their inclusion within the advantaged side of the distribution.

The second set of outcomes, which includes incarceration, all-cause mortality, and life expectancy, follows a Black-White-Asian ordering. Cultural normative interpretive strain again arises from the bottom placement of Black Americans, while structural frameworks are immediately strained because Asian Americans, rather than White Americans, occupy the most advantaged position. Triadic comparison further amplifies this strain. In incarceration, White Americans experience a rate of approximately 337 per 100,000 compared to 1,826 per 100,000 for Black Americans and 141 per 100,000 for Asian Americans. These rates place White Americans substantially closer to Black American disadvantage than to Asian American advantage. A similar pattern appears in all-cause mortality. White mortality rates are markedly higher than Asian mortality rates and substantially closer to Black mortality rates, with life expectancy exhibiting the same directional tendency. Across both sets, the interaction of position and rate demonstrates that triadic comparison generalizes and intensifies evaluative strain rather than resolving it.

Then it might be said that, thus far the analysis has only selected variables where Black is disadvantaged and where White and Asian are advantaged. What about outcomes such as measures of family stability, community cohesion, political participation, or middle-class attainment (cf. Pattillo, 2021) where Black Americans perform comparatively better? These outcomes would counterbalance cultural deficit interpretations anchored in poverty or education alone. Similarly, what about outcomes such as workplace discrimination or political underrepresentation (Lee and Sheng 2024; Wong and Ramakrishnan, 2023), in which Asian Americans appear disadvantaged or burdened? These outcomes preserve the disadvantaged-advantaged contrast central to structural interpretation. The remaining sets of rows in Table 4 show outcomes mapping onto White-Black-Asian, Asian-Black-White and, Asian-White-Black orderings, respectively.

With these sets of outcomes there are several cases in which cultural normative strain is partially alleviated because Black Americans are positioned closer to the higher pole not only in rank but also in rate. This pattern is visible in outcomes such as serious psychological distress, national belonging, and self-esteem. For example, in serious psychological distress, Black Americans report a rate of 11.4 percent, which is closer to Asian Americans at 10.2 percent than to White Americans at 14.2 percent. In national belonging (where lower represents greater bellowing), Black Americans report 27 percent which is closer to the White rate of 7 percent than the 64 percent rate for Asian Americans. In self-esteem and union representation Black Americans are on the higher side compared to either White or Asian. In each of these cases, Black Americans is rate-wise proximate to or exceed the higher pole, disrupting deficit-based interpretation. Thus, these triadic cases then work with cultural frameworks. At the same time, these configurations are comparatively rare within the full set of outcomes examined, limiting their ability to counter the dominant pattern observed across the full range of domains.

Structural frameworks find markedly less support in these final outcome sets. Voter turnout stands out as the only variable that aligns cleanly with the structuralist framework once triadic comparison and rate proximity are considered. In voter turnout, White Americans exhibit the highest participation rate at 71 percent, followed by Black Americans at 63 percent and Asian Americans at 60 percent. Crucially, Black Americans’ turnout rate is closer to Asian Americans than to White Americans, preserving a disadvantaged–advantaged contrast in which White Americans retain the advantaged position while Black Americans remain relatively constrained. This configuration allows structural interpretation to proceed with a POC vs. White comparison without reassigning advantage away from White Americans or redefining dominance. By contrast, the other outcomes in these sets strain structural explanation. In national belonging, Black Americans report 27 percent compared to 64 percent for Asian Americans and only 7 percent for White Americans, pushing Black Americans toward advantage rather than disadvantage. In self-esteem and union representation, Black Americans occupy the highest numerical position. In these cases, structural frameworks are strained because advantage either accrues to Asian Americans or is pulled toward Black Americans, severing the expected link between historical dominance and superior outcomes.^7^

## 2 Discussion: normative interpretive strain “repair responses”

Seen in this light it is then possible to situate various developments in the literature as functioning, at least in part, as downstream “repair responses” to normative interpretive strain. These responses have the effect of redirecting the evaluative implications of aggregate outcomes. The recurrent emergence of such repair responses suggests that debates over “difficult to interpret” racial disparities are in large part debates over the normative admissibility of placing particular racial groups into particular evaluative positions. While some have argued that deficiency may at times be the more accurate characterization, structural interpretations often encounter normative interpretive strain once an “undesirable attribute gets attached to a canonically victimized target.” (Martin, 2016, 117). Here we identify three such responses across both frameworks: heterogeneity (subdivides the groups), compositional selectivity (adjusts the comparison), and multiplicity (allows for multiple positions). After describing each one we explain why the repair is only partial.

### 2.1 Heterogeneity

One prominent strategy involves emphasizing within-group heterogeneity. The logic is that aggregate racial disparities obscure important subgroup variation, such that, based on empirical outcome rates, only some subgroups within a broader racial category occupy the “deficient” or “advantaged” side of the disparity while others must be classified differently. In other words, not “all” ethnic subgroups within a racial group belong on the negative “deficit” or negative “advantaged” side. In this sense, heterogeneity arguments seek to weaken the interpretive force of broad racial categories themselves by suggesting that aggregate racial rankings are overly coarse and analytically misleading. Rather than treating “Black,” “White,” or “Asian” populations as internally uniform blocs, these approaches argue that the meaningful unit of analysis is often the ethnic subgroup, where substantial variation in education, income, poverty, health, or mobility outcomes becomes visible. The broader implication is that racial disparities may appear less normatively problematic once disaggregated, because the evaluative meanings attached to the aggregate category no longer apply uniformly to all constituent subgroups.

Ethnic heterogeneity arguments, for example, highlight variation within Black populations by focusing on relatively high-performing groups, such as Nigerians in education or income (Sakamoto et al., 2021). These outcomes are used to temper generalized cultural “deficit” and structural disadvantage implications in regards to Black Americans. More recent heterogeneity analysis shows substantial socioeconomic heterogeneity within the White population. Some White ethnic groups such as Romanians exhibit incomes that are comparable to or worse than those of Black Americans (Sakamoto et al., 2026). These outcomes are used to temper claims of uniform White “advantage.” Other studies have placed emphasis on heterogeneity within Asian populations. Tri-racial hierarchy theories position Black groups at the bottom, White groups at the top and split Asian groups between the top, middle and bottom positions (Bonilla-Silva, 2004). Here the argument is that certain Asian subgroups such as Cambodian, Hmong, or Laotian Americans are disadvantaged compared to others (ibid: see also Drouhot and Garip, 2021). Here the aim is to temper claims of Asian American “advantage.”

In this sense heterogeneity is used in some cultural theories to challenge claims of uniform Black “disadvantage” and uniform White “advantage,” and by some structural theories to challenge claims of uniform Asian “advantage.” In this respect, it is true that not all subgroups fit the aggregate profile. The analytical problem is that once analysis shifts to the ethnic group level, then the research project and the analytical process shift away from the concept of “race”; that is, the research focus now has shifted after-the-fact but often does not lead to the reframing of research projects and the analytical processes. Further, subgroup comparisons must be applied consistently across all racial groups. Disaggregating one racial group into ethnic subgroups while leaving others aggregated introduces an asymmetry in the analysis (see Sakamoto et al., 2026 on this point). The tri-racial hierarchy approach, for example, treats class variation as only salient within the Asian American population.^8^ If applied consistently, the analysis would no longer compare broad racial categories such as Asian, Black, and White, but would instead expand into twenty or more distinct ethnic subgroups. Even under this more granular framework, the groups will have to be comparatively ordered. Depending on the framework used, some ethnic subgroups would continue to occupy the “deficient” side of the contrast, while others would occupy the “advantaged” side. Heterogeneity and sub-group analysis does not resolve the broader issue of group problematization within either cultural or structural frameworks. Instead, heterogeneity analysis changes *who is problematized* from a racial group to a smaller set of ethnic subgroups.

### 2.2 Compositional selectivity

Another repair strategy, used primarily within structural frameworks, challenges the initial placement of racial groups within evaluative contrasts by arguing that the introduction of additional compositional or demographic controls would alter the groups’ relative positioning within the disparity itself. The claim is not that the aggregate disparity disappears, but that once factors such as immigration selectivity and nativity are taken into account, the apparent placement of groups as “advantaged” or “disadvantaged” changes. In other words, the identification of “who” is “advantaged” or “disadvantaged” changes. Such arguments are most commonly invoked when a particular racial group occupies a normatively difficult position.

Immigration selectivity arguments, for example, maintain that compared to other racial groups Asian Americans disproportionately descend from positively selected immigrant populations with comparatively high levels of education and economic resources. Controls for nativity, immigrant generation, or parental socioeconomic status are held to reduce apparent Asian American advantage (Acciai et al. 2015; Tran, 2024; Zhou and Lee 2017). Similar arguments as to why Asians are healthier than other groups also invoke nativity with the arguments that groups with higher percentage foreign-born are healthier (see also Hummer and Chinn, 2011 on Latinos).

In the case of immigrant selectivity, controls for nativity may move Asian American outcomes closer to, or even below, White outcomes on some measures. It then appears that Asian American is disadvantaged and White is advantaged. Yet this repair strategy remains largely organized around the Asian-White dyad, often dropping Black Americans from the comparison altogether. The implicit issue, therefore, is not simply whether Asian Americans remain advantaged relative to Whites once the appropriate controls are introduced. The question is whether such controls mean that Asian Americans fall closer to the “disadvantaged” side occupied by Black Americans within the broader disparity structure. Across most outcomes, however, even after compositional selectivity adjustments, Asian American, rates remain substantially closer to White than to Black outcomes even after adjustment. In this sense the underlying normative interpretive strain for structural frameworks therefore persists.^9^

### 2.3 Multiplicity

Another approach is to combine evaluative positions both across and within frameworks, as well as across contexts such as different outcomes, places, or periods of time. In other words, racial groups can simultaneously occupy multiple evaluative positions. Across frameworks, groups may be represented, for example, as culturally meritorious yet structurally disadvantaged or culturally deficient yet structurally constrained. Within frameworks themselves, the meaning of deficit-merit and advantage-disadvantaged may be reinterpreted; groups may also be represented as simultaneously structurally advantaged and disadvantaged across different outcomes, as occupying positions that are culturally deficient in one respect yet culturally adaptive in another, as structural in one era and cultural in another. Alternatively, the distinction between culture and structure itself collapses, such that culturally valued traits are understood as institutionally rewarded and embedded within relations of power rather than as autonomous group characteristics. The effect is not to eliminate asymmetrical positioning, but to multiply and partially offset the meanings attached to it. In this sense, the negative evaluative implications associated with occupying either side of a disparity become partially neutralized. Lower positions need not signify straightforward cultural deficiency, while higher positions need not signify uncomplicated structural advantage.

A*dvantage in one area, disadvantage in another:* Racial triangulation theories, for example, explicitly position Asian, Black, and White groups within multidimensional rather than strictly linear hierarchies (Kim, 1999; Kim, N. Y., 2022). In this formulation, Asian Americans may be represented as relatively advantaged in relation to Black Americans on socioeconomic outcomes while simultaneously remaining civically excluded, foreignized, or subordinate relative to Whites. Here, relative advantage on one dimension is combined with disadvantage on another, such that groups occupy multiple evaluative positions simultaneously. A*dvantage is a form of disadvantage:* “bamboo ceiling” arguments, maintain that Asian Americans achieve comparable income and wealth relative to Whites only because they must over-invest in education and exert substantially greater effort to obtain similar returns (Tran et al., 2019). Here, apparent Asian American advantage is recast as a distinctive form of disadvantage associated with unequal returns, positive stereotyping, or psychosocial burden (Walton and Truong, 2023; Wu et al., 2018). *Deficit as merit;* Parallel moves also appear in accounts that reinterpret Black “deficit” as adaptive or understandable under conditions of structural constraint through narratives of resilience, cultural creativity, or strategic response to inequality (Keyes, 2009; Small et al., 2010).

Multiplicity arguments typically redefine the evaluative dimension under consideration. The problem, however, is that these reformulations are typically selectively applied to the racial group whose positioning led to evaluative interpretive strain. Both racial triangulation and bamboo ceiling arguments, for example, largely focus on the Asian-White comparison while dropping the Black-Asian one (Davies, 2022; Kim, C. J., 2022). The net result is a theory in which the group on the bottom and the top are treated as largely equally if differentially structurally disadvantaged (Wilkes and Karimi, 2025a, 2025b, 2026; Sexton, 2010; Kim, 2024). Insofar as White Americans are theoretically defined as dominant when occupying an empirically intermediate or even lower position then dominance becomes progressively detached from comparative outcome position itself. Cultural adaptation arguments attempt to neutralize deficit-oriented interpretations attached to comparatively poor outcomes. Yet doing so destabilizes the original merit-deficit contrast underlying cultural explanation. If comparatively poor outcomes are treated merely as non-deficient, the contrast weakens into one between merit and non-deficit. If they are reinterpreted positively through resilience, creativity, or adaptation, the contrast risks collapsing into competing forms of merit or competence. The group at the top cannot be “meritorious” if the group at the bottom is not “deficient.” In either case, the evaluative distinction between comparatively high and low positions becomes increasingly difficult to stabilize.

## 3 Conclusion

This paper shifted attention from the causes of racial disparities to the interpretive frameworks through which disparities are rendered meaningful once observed. Rather than treating disparities as neutral empirical differences awaiting explanation, the analysis showed that cultural and structural frameworks organize interpretation through distinct evaluative grammars that assign meaning to higher and lower group positions prior to causal explanation itself. Cultural frameworks tend to organize disparities through a deficient-meritorious contrast (Auletta, 1982; Lewis, 1966; Murray, 1984), while structural frameworks organize them through a disadvantaged-advantaged contrast (Bonilla-Silva, 1997; Feagin and Elias, 2013; Tilly, 1998). In both cases, disparities require asymmetrical evaluative positioning. The concept of “normative interpretive strain” was introduced to describe the tension that emerges when empirical outcomes place particular racial groups into evaluatively difficult or problematized positions within these frameworks. Cultural interpretations encounter normative interpretive strain when Black Americans repeatedly occupy the lower side of disparities because such placements reactivate deficit-oriented interpretations (Auletta, 1982; Lewis, 1966) that are now widely rejected. Structural interpretations encounter normative interpretive strain when Asian Americans occupy relatively advantaged positions or when White Americans occupy intermediate or comparatively disadvantaged positions, thereby disrupting the expected alignment between advantage and historical dominance (Bonilla-Silva, 2004; Kim, 1999). The significance of these tensions lies not in the outcomes themselves or in whether the frameworks are capable of explaining them, but in the normative difficulty of assigning particular racial groups to the evaluatively problematized side of the contrast.

The dyadic and triadic analyses showed that interpretive strain depends on how empirical group orderings align with the evaluative contrasts embedded within cultural and structural frameworks. In the dyadic analysis, Black-White disparities in poverty, education, and income aligned more readily with structural interpretations because Black Americans occupied the disadvantaged position and White Americans the advantaged one. The same outcomes strained cultural interpretations by pulling toward deficit-oriented readings of Black disadvantage. White-Asian comparisons produced the opposite pattern: Asian Americans’ comparatively favorable educational and income outcomes fit more easily within cultural interpretations, while structural interpretations encountered strain because advantage no longer aligned clearly with historical dominance. Black-Asian comparisons strained both frameworks simultaneously. The triadic analysis intensified these tensions. In poverty, Asian Americans occupied an intermediate position but remained much closer to White than Black Americans, while in education and income Asian Americans occupied the highest position outright and White Americans often appeared closer to Black Americans than to Asian Americans. Once all three groups were analyzed simultaneously, the apparent division of analytic labor between cultural explanations for White-Asian disparities and structural explanations for Black-White disparities became increasingly difficult to sustain.

The concept of “normative interpretive strain” helps engage a range of theories as operating, at least in part, as “repair” strategies. These repair strategies attempt either to reposition racial groups within the disparity or to redefine the evaluative meaning attached to the positions they occupy. Heterogeneity arguments shift attention from racial groups to ethnic subgroups in order to weaken aggregate claims of deficiency or advantage, yet asymmetrical positioning reappears at the subgroup level (Bonilla-Silva, 2004; Drouhot and Garip, 2021; cf. Sakamoto et al., 2026). Selectivity and compositional arguments introduce controls intended to alter apparent group placement within disparities, yet the broader comparative ordering frequently remains intact (Acciai et al. 2015; Tran, 2024). Multiplicity arguments reinterpret the meaning of advantage and deficit themselves through adaptation, resilience, exclusion, or multidimensional positioning (Kim, 1999; Walton and Truong, 2023; Keyes, 2009). In effect repair strategies seek to reposition racial groups within disparities or to redefine the evaluative meanings attached to the positions they occupy, whether through subgroup differentiation, compositional adjustment, or multidimensional reinterpretations of advantage and disadvantage.

These repair strategies do not eliminate normative interpretive strain because they cannot remove the basic requirement that comparative analysis place groups on different sides of an evaluative contrast. Instead, they work by creating “escape” hatches for the particular racial group whose empirical position creates strain within the framework. Heterogeneity arguments shift attention from racial groups to ethnic subgroups, but subgroup comparisons still require that some groups occupy the negative deficient or advantaged side of the contrast. Selectivity and compositional arguments attempt to reposition groups through controls and adjustment, yet the broader comparative ordering often remains intact. Multiplicity arguments redefine particular groups as simultaneously advantaged and disadvantaged across different dimensions, but the new categories are usually applied selectively to the normatively strained group rather than consistently across all groups in the comparison. Across these approaches, the underlying implication is often that this particular group should not occupy the problematized position within the disparity. Yet the implications for the remaining groups are frequently left unexamined, including the fact that once one group is removed from the problematized position, another group must still occupy it. If all groups were treated as simultaneously meritorious and deficient, advantaged and disadvantaged, the distinction between the two sides of the normative contrast would become difficult to maintain.

Comparative racial analysis cannot proceed without attaching evaluative meanings to group position. Whether disparities are interpreted through the language of culture or structure, groups become positioned as deficient or meritorious or, disadvantaged or advantaged. Normative interpretive strain is therefore not a failure of theory, data, or methodological rigor. It is a recurring consequence of how racial disparities are rendered sociologically intelligible. Someone has to be “the problem.” This is why outcomes that appear manageable in one comparison often become difficult once additional groups or outcomes are introduced. The problematization often becomes more immediately visible. The evaluative implications attached to group position become harder to stabilize across multiple comparisons at once. The persistence of repair responses further suggests that this tension can be managed, displaced, or reformulated, but never fully eliminated. The broader implication is therefore not simply that some racial disparities are more difficult to explain than others. It is that comparative racial analysis itself continually generates pressure to stabilize the evaluative meanings attached to racial group differences while avoiding the implications of assigning particular groups to the problematized side of the disparity.

## Data Availability

The original contributions presented in the study are included in the article/supplementary material, further inquiries can be directed to the corresponding author.

## Footnotes

1 We note the historically specific U.S. context of the selected groups and the associated meanings attached to race. In this context, there is a similar tension in relation to other gaps and contrasts including Black-Indigenous (Garba and Sorentino, 2020) and White-Latino (Abraído-Lanza et al., 1999). Elsewhere, comparative analysis may still position groups asymmetrically along higher and lower outcome distributions, though normative interpretive strain may be less likely where such evaluations are more socially or politically stabilized.

2 As (Iceland and Silver, 2024: 1078) note, when a field converges around a single ideological perspective “scientific output that deviates from that field’s ideology gets scrutinized more severely than output that conforms to it resulting … in a skewed understanding of the social processes that generate the empirical patterns in question.”

3 Applied to racial disparities in poverty, early cultural accounts then attributed Black disadvantage to deficit-oriented cultural traits such as “fatalism, dependency, and helplessness” (Lewis, 1966, p. 23) qualities held as acting as barriers to mobility. Later work in this tradition also framed such responses as present though qualified them as caused by structural joblessness and social isolation in inner-city contexts (Wilson, 1987). Subsequent work also focused on deficits including “conspicuous consumption” (Moav and Neeman, 2012), single-parent family structure (Amato and Maynard, 2007), devaluation of formal education (Downey, 2008; Ogbu, 2004), as well as an absence of conventional work ethic (Office of Policy Planning and Research (Moynihan) and Daniel, 1965). In quantitative research, culture has often been operationalized through variables such as educational attainment (Hunt, 2004), single-mother headed family structure (Baker and O’Connell, 2022), and bankruptcy and excessive spending (Herring and Henderson, 2016).

4 Some examples of variables used to reflect structural arguments include whether the respondent is in the bottom and top 20% of the income distribution (Herring and Henderson, 2016), county percent enslaved in 1860 (Baker and O’Connell, 2022) and geographic context (Baker et al., 2022).

5 Links to the sources for this and all subsequent data are provided in Table A1.

6 Insofar as the disparity between the Black and White groups is treated as caused by factors such as education, family structure, and spending habits then the causes of the disparity are essentially treated as non-racial, for example, through formulations in which education → disparity (Thomas, 2000). This formulation treats such attributes as exogenous, rather than as outcomes shaped by racialized processes (Williams, 2019). Thus, it is not simply that education is the cause of the disparity. Instead, racism is also the cause of differing levels of education (Merolla and Jackson, 2019) as well as returns to education via employment discrimination, as captured in formulations such as racism → education disparity → economic disparity (Pager and Shepherd, 2008).

7 Extending the analysis to additional racialized groups is unlikely to resolve the normative interpretive strains identified here and would instead redistribute them. American Indian and Alaska Natives have rates on many outcomes that are typically closer to, or even below Black American (Blue Bird Jernigan and Maudrie, 2025). Latinos, by contrast, constitute a group that lacks historical dominance and remains subject to racialized constraint similar to, but different from Black Americans (cf. Vargas et al., 2025). Yet Latinos have relatively favorable health outcomes, including mortality and life expectancy rates that equal or exceed those of White Americans (Fenelon, 2013), despite lower average socioeconomic status (Abraído-Lanza et al., 1999; Markides and Coreil, 1986). On other indicators of criminal justice contact, including incarceration rates and police use of lethal force, Latino outcomes are consistently closer to those of White and Asian populations than to those of Black Americans (Wilkes and Karimi, 2023). This configuration creates interpretive strain within cultural frameworks because Black Americans may then occupy a relative deficit position vis-à-vis Latinos, despite such comparisons rarely being foregrounded analytically. Similarly, for the structural framework, any evidence of Latino advantage produces the same kind of normative interpretive strain as Asian advantage, since such evidence requires explaining favorable outcomes for a non-dominant group.

8 Bonilla Silva for example, uses class to order Asian ethnic subgroups as either “white” (some Asians); “honorary white” (Japanese, Korean Americans) and collective Black (Vietnamese Hmong Americans). Yet all White groups are treated as “White” regardless of class and all Black groups are treated as “collective black” regardless of class.

9 We note that in the case of age, the argument is that in certain cases where the White population has worse health outcomes than others such as Black Americans this is because older people tend to have worse outcomes and the White population is older (Arolas et al., 2023). For certain specific health outcomes age standardization does substantially reduce, and in some cases reverse, apparent White disadvantage (ibid).

## Appendix

**Table A1.** Outcome data sources.

Outcome	Source	Table
Poverty %, 2023	Poverty in the United States: 2023	A3
Education % (college grad), 2023	https://data.census.gov/table/ACSST1Y2023.S1501?q=S1501:+Educational+Attainment	S1501
Income median $, 2024	Income in the United States: 2024	A2
Poverty children %, 2023	Poverty in the United States: 2023	A3
Home ownership %, 2024	Housing Vacancies and Homeownership - Annual Statistics	22
Usual place of health care %, 2024	NHIS-Adult Summary Health Statistics	–
Wealth median net worth, 2022	https://www.census.gov/library/visualizations/interactive/assets-and-debts.html	–
Manag /prof occ. %, 2024	Employed persons by occupation, race, Hispanic or Latino ethnicity, and sex	10
Unemployment rate %, 2023	https://www.bls.gov/opub/reports/race-and-ethnicity/2023/	Chart 4
No health insurance, % 2024	NHIS-Adult Summary Health Statistics	–
Life expect. at birth years, 2023	https://www.cdc.gov/nchs/data/nvsr/nvsr74/nvsr74-06.pdf	Fig 2
All cause mortality per 100K age adjusted, 2023/24	https://www.ncbi.nlm.nih.gov/books/NBK619358/table/vsrr039.t1/?report=objectonly	1
Incarc. per 100k, 2012-2022	https://bjs.ojp.gov/document/p22st.pdf	13
Serious psychological distress crude rate %. 2023	NHIS-Adult Summary Health Statistics	–
Smoking during pregnancy crude rate %, 2023	NHIS-Adult Summary Health Statistics	–
Mortality from unintentional injuries % age adjusted, 2023	Dwyer-Lindgren, Laura, et al. "Life expectancy by county, race, and ethnicity in the USA, 2000–19: a systematic analysis of health disparities." *The Lancet* 400, no. 10345 (2022): 25-38.	1
National belonging asked where are you from? %, 2021	Wong, Janelle S., and Karthick Ramakrishnan. "Asian Americans and the politics of the twenty-first century." *Annual Review of Political Science* 26, no. 1 (2023): 305-323.	Fig 4
Voter turnout %, 2020	Large Racial Turnout Gap Persisted in 2020 Election | Brennan Center for Justice	–
Senior executive service rep/l.f. share %, 2018	https://www.opm.gov/policy-data-oversight/data-analysis-documentation/federal-employment-reports/reports-publications/ses-summary-2014-2018.pdf	8
Self-esteem effect size vs White baseline, 2002	Twenge, Jean M., and Jennifer Crocker. "Race and self-esteem: meta-analyses comparing whites, blacks, Hispanics, Asians, and American Indians and comment on Gray-Little and Hafdahl (2000)." (2002): 371.	Fig 1
Union representation among employed workers %, 2024	Table 1. Union affiliation of employed wage and salary workers by selected characteristics - 2024 A01 Results	1
